# Restriction Profiling of 23S Microheterogenic Ribosomal Repeats for Detection and Characterizing of* E. coli* and Their Clonal, Pathogenic, and Phylogroups

**DOI:** 10.1155/2015/562136

**Published:** 2015-12-22

**Authors:** Parvathi Jayasree Rajagopalan Nair, Sunita Singh

**Affiliations:** School of Biotechnology & Bioinformatics, D. Y. Patil University, Plot 15, Sector 50, CBD Belapur, Navi Mumbai, Maharashtra 400614, India

## Abstract

Correlating ribosomal microheterogenicity with unique restriction profiles can prove to be an efficacious and cost-effective approach compared with sequencing for microbial identification. An attempt to peruse restriction profiling of 23S ribosomal assemblage was ventured; digestion patterns with* Bfa* I discriminated* E. coli* from its colony morphovars, while* Hae* III profiles assisted in establishing distinct clonal groups. Among the gene pool of 399 ribosomal sequences extrapolated from 57* E. coli* genomes, varying degree of predominance (I > III > IV > II) of* Hae* III pattern was observed. This was also corroborated in samples collected from clinical, commensal, and environmental origin. K-12 and its descendants showed type I pattern whereas* E. coli*-B and its descendants exhibited type IV, both of these patterns being exclusively present in* E. coli*. A near-possible association between phylogroups and* Hae* III profiles with presumable correlation between the clonal groups and different pathovars was established. The generic nature, conservation, and barcode gap of 23S rRNA gene make it an ideal choice and substitute to 16S rRNA gene, the most preferred region for molecular diagnostics in bacteria.

## 1. Introduction

The need of rapid assay with low detection limits has reverberated genotypic techniques to gain momentum in diagnostic field which not only revolutionized but enhanced the authentication process. Within a short span, the evolving concepts and strategies for molecular detection have resulted in the shift of “gold standard” from being DNA-DNA hybridization (DDH) to currently Nucleic Acid Amplification Techniques (NAATs) and gradually towards Average Nucleotide Identity (ANI) [1–4]. Among the NAATs, Polymerase Chain Reaction (PCR) based on amplification of 16S rRNA region [5, 6] followed by the sequencing of amplicon forms a simple yet efficient rationale for bacterial barcoding [7]. Basis for targeting this gene segment is reasoned by the ubiquitousness in bacterial kingdom to multiple ribosomal RNA copies/numbers,* rrn* [8, 9] providing sensitivity in PCR reaction. Another remarkable feature of ribosomal appendage is the degree of conservation and heterogenicity, with the nonvariable, slow evolving regions preferred for the identification and variable regions being the choice for phylogenetic studies [10, 11]. While designing universal or specific primers against these stretches, parameters like primer specificity, hybridization efficiency, and amplicon incongruity arising due to microheterogeneity within* rrn* copies must be taken into consideration [12, 13]. These precautionary measures can help in limiting the pitfalls of molecular assay and help to winnow the ingrained information regarding the usability and effectiveness of primers for identification. An alternative to this scenario is to exploit inter- and intragenic ribosomal microheterogeneity for developing Single Nucleotide Polymorphism (SNP) based discriminative methods. The logic behind this stratagem is to develop broad range primers against microdiverse regions of the targeted stretch which encompass distinct restriction profile characteristic to each organism(s) under study [14].

This current study is an endeavor to entitle 23S rRNA gene as substitute to* de facto* barcode, 16S rRNA gene using amplification ensued by restriction digestion instead of sequencing, making it a cost-effective approach. The exemplification of the proposed method was carried out to detect and differentiate* E. coli* (candidate) from* E. aerogenes*,* K. pneumoniae*, and* C. koseri* (noncandidates). The above choice was finalized after considering the following: (i) all the selected microbes, both candidate and noncandidate, are Gram negative, lactose positive microbes of *γ*-Enterobacteriaceae family [15]; (ii) all are urinary tract infection (UTI) pathogens with* E. coli* being reported as the major causative factor [16–18]; and lastly (iii) all exhibit similar colony semblance and any false positive and negative biochemical characterization resulting in misinterpretation with candidate strain.

In spite of being the model representative of bacterial kingdom,* E. coli* is also acknowledged for its omnipresence and the capability of switching from good to bad microbe. Pathogenic* E. coli* are classified into aggressive, invasive, pathogenic, hemorrhagic, and toxigenic which differs in their virulence genes acquired and the type of pathogenicity [19]. Comparative study of commensal and pathogenic strains of the same has shown a rather surprising result of fewer pathovar specific sequences evincing* E. coli* as a genera list [12]. Sharing of 40% of core genomes among both virulent and nonvirulent strains indicates clonality [20]. This conceptualizes an open pangenome having genetic reservoirs to allow gene acquisition for evolving into pathogenic ones [21].

Besides isolates of* E. coli* present in nature, the routinely used nonvirulent lab strains are K-12, B, W, Crooks, and C with all being fecal in origin except for strain W (soil isolate) [22]. Method of rapid identification of K-12 strains using mutations in O-antigen gene cluster [23] and determination of lab strains lineage using multiplex primers [24] have been reported. Studies on the lineages of well-established* E. coli* lab strains across the globe have shown that most strains are derived from either K-12 or B [25, 26]. Genomes of these supposed to be parental strains show 99% genetic identity with exceptions to IS elements and flagellar genes [27]. Phylogenetic classification of* E. coli* strains into A, B1, B2, D, and E groups using Multiloci Enzyme Electrophoresis, MLEE [28], and triplex [29] has given insights into the concept of clonality in* E. coli*. A comparative analysis of MLEE and random amplification, restriction profiling against 16S-23S intertranscribed region, and ribotyping have verified that horizontal gene transfer mechanisms do not disrupt organization of clonal population [30].

No study till date has correlated Amplified Ribosomal DNA Restriction Analysis (ARDRA) of a core segment in 23S rRNA gene against the pangenome and tried to establish a link between phylogroups, pathogroups, and restriction groups.

## 2. Materials and Methods

### 2.1. Bacterial Strains and Culture Conditions

Authentication of the molecular assay in screening and identifying the candidate organism was executed into parts, one with standard strains of* E. coli* and the other with 25 isolates each from fecal, clinical, and environmental origin. Lyophilized strains were procured from Microbial Type Culture Collection (MTCC), Institute of Microbial Technology (IMTECH, Chandigarh, India), for both identification and clonality studies (Table 1).

Five different sets of fecal droppings from rat, chicken, pigeon, rabbit (pet shops, Crawford market, Mumbai, India), and humans (Excel Pathology Lab, Vashi, Navi Mumbai) were considered for isolation of commensal samples. The strains were revived in nutrient broth at 37°C. For environmental strains, water sample from ten local water bodies lying in and around the parent institute was utilized (areas with hospitals or health care centers in the vicinity were avoided to prevent cross-contamination with clinical samples). For isolating* E. coli* of clinical origin, urine samples from twenty-five patients suffering from UTI (Excel Pathology Lab) were considered.

The samples were processed within 24 hrs of collection (either in zip log bags or in sterile glass bottle) on MacConkey agar (MAC). The distinct pink colonies were restreaked onto both MAC and Eosin Methylene Blue (EMB) agar, respectively [31]. Isolates giving discrete colony morphology with indeterminate greenish sheen were selected and evaluated for the false positive and negatives by both microbiochemical and molecular assays. Each of the selected colonies was picked with sterile wooden toothpicks and transferred to five mL of nutrient broth and incubated overnight at 37°C. These samples were further characterized [32] by IMViC; 1 mL was utilized for DNA isolation and the remaining was used for preparing glycerol stock (25% glycerol) for future reference.

### 2.2. Genome Database and Microheterogenicity Analysis

**Figure 1:**
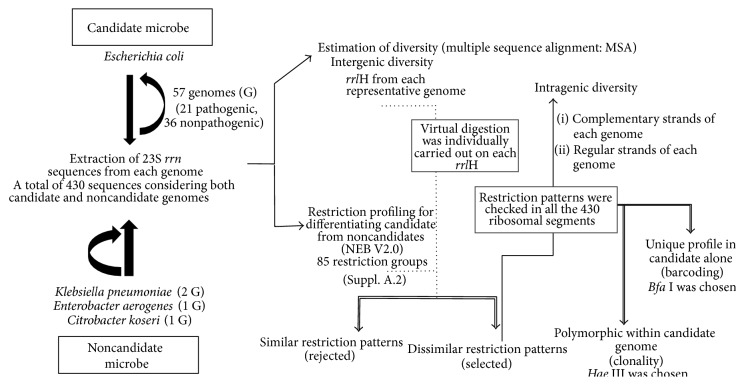
Schematic representation of methodology for detecting barcode and estimating clonality. The primers are designed against the regions that flank these restriction sequences which gives unique bands revealing identification or clonality.

The aboriginal step involved the aggregation of Genbank genome sequences of all the strains that were considered for this study from the public data repository, National Centre for Biotechnology Information (NCBI). The data pool (until 11 April 2013) comprised an assemblage of 61 completed genomes (Supplementary Data A.1 in Supplementary Material available online at http://dx.doi.org/10.1155/2015/562136) with 57 of candidate strains (*E. coli*) and noncandidate strains (*K. pneumoniae*,* C. koseri*, and* E. aerogenes*). The following steps were executed to analyze the microheterogenicity (Figure 1) which can directly assist in evaluating the electability of 23S rRNA gene as an ideal barcode.

Two strains, one from the candidate and one from noncandidate genome (*E. coli* str. K-12 MG1655 (NC_000913.2),* C. koseri* ATCC BAA-895 (NC_009792.1),* E. aerogenes* (NC_015663.1), and* K. pneumoniae* subsp*. pneumoniae* MGH 78578 (NC_009648.1)), were selected and assigned as reference strain.

For studying intergenic variability among the candidate and noncandidate strains, the* rrs*H (16S RNA gene) and* rrl*H (23S rRNA gene) sequence on the regular strand (reference strains) were extrapolated and multiple sequence alignment (MSA) using ClustalW [33] was executed. Total mismatch was enumerated by the addition of number of mismatches and sequence gaps introduced during the “line-up” of two or more sequences. The mismatch percentage was calculated by dividing the total mismatches seen across the four strains by the average base pair (bp) for both 16S and 23S* rrn* gene, respectively.

For estimating the intragenic variability, all the ribosomal segments from 57* E. coli* genomes were extracted (7 copies/genome); complementary and regular strands within each strain were segregated and the percentage mismatches for each of the genomes were determined.

### 2.3. Virtual Restriction Profiling

Virtual restriction profiles using NEB V2.0 [34] with 85 restriction groups (Supplementary Data A.2) were performed for a total of 430 ribosomal segments that included 399 from 57* E. coli* genomes and 7, 8, and 16* rrn* sequences from C.* koseri*,* E. aerogenes*, and* K. pneumoniae* strains, respectively. Among the 14 common cutters groups (Supplementary Data A.3),* Bfa* I (Supplementary Data A.4) was selected as the enzyme for barcoding as it gave conserved and distinct pattern across all the 399* rrn* sequences (not shown) in differentiating the candidate from the noncandidate strains. The uniqueness and position of the cuts sites for giving unique bands on agarose gel electrophoresis were also a lead reason considered for finalizing this enzyme.* Hae* III displaying polymorphism pattern with 23S rDNA segments of candidate microbe was finalized for diversity studies (Figure 1).

### 2.4. Primers and Restriction Enzymes for Addressing Identity and Clonality

Sequence of* rrl*H of* E. coli* K-12 str. MG1655 was used as the template for designing broad range primers using Primer-BLAST [35]. In conjunction with parameters like GC content, *T*_*m*_ value, and the amplicon size number of restriction sites within the amplicon and the size of fragment after digestion were kept as the benchmark for finalizing primers. There are two primer sets: 23S P1_880_ [Probe 31778738] was selected with* Bfa* I restriction digestion (Table 2) demarking the candidate and 23S P2_682_ [Probe 31781046] (Table 3) with polymorphic* Hae* III restriction sites was selected for addressing clonality. Possibility of self-priming and primer-dimer formation of each primer was checked using free web tools {Oligocal (http://www.simgene.com/OligoCalc), OligoEvaluator (http://www.sigmaaldrich.com/technical-documents/articles/biology/oligo-evaluator.html), and multiple primer analyzer tool (http://www.thermoscientificbio.com/webtools/multipleprimer/) maintained by SimGene, Sigma, and Fermentas, resp.}.

BLAST genome (Supplementary Data) assisted in verifying the conservation and specificity of primers, amplicon size,* Bfa* I recognition sites, and region of hybridization across the genome of 931 bacterial families. After omitting the nonannotated sequences, all of the completed whole genome data aggregated under phyla Actinobacteria, Bacteroidetes, Chlamydiae, Cyanobacteria, Firmicutes, Proteobacteria, and Spirochaetae of the bacterial kingdom were considered for the analysis.

### 2.5. DNA Isolation, Amplification, and Restriction Digestion

DNA isolation was performed according to Parvathi and Singh [36]. The reaction soup comprised 2 *μ*L of supernatant (DNA), 0.5 *μ*M of each forward and reverse primer, 1x of assay buffer (10 mM Tris-HCl, pH 8.8, 50 mM KCl), MgCl_2_ (1.5 mM), dNTP mix (800 *μ*M), and 0.5 U/*μ*L Taq polymerase (Kappa Systems, KK5004). Standard genomic DNA of* E. coli* K-12 (Merck) and nuclease-free water were used as positive and negative control. The reactions were carried out in a thermal cycler (Bioer) with the program set for 25 cycles comprising denaturation (95°C), annealing (68°C), and extension (72°C) for 30 s each. 3 *μ*L of the PCR product was digested with 2 U of respective enzyme (Fermentas, Thermo Scientific) in 5 *μ*L reaction mix at 37°C for two hours. The amplicons and their restriction products were analysed by agarose gel electrophoresis (AGE) at 5 V/cm in 2% and 2.5% agarose gels, respectively, each containing 0.5 *μ*g/mL of EtBr. The gels were viewed and photographed in BioImaging Systems (Syngene).

## 3. Result and Discussion

### 3.1. Genetic Diversity within 23S Ribosomal Repeats for Identification of* E. coli*

23S rRNA gene showed 0.87% intergenic variations compared with 16S rRNA within the reference genomes (Supplementary Data A.5). The result seemed obvious due to the increased frame size of the latter but this also encouraged perusing microheterogenicity analysis of the largest ribosomal segment for further approach. The sizes of target segments varied from 2903 to 2907 bp with maximum gene length observed in ATCC8739 (Crook's strain) accounting for 2970–2985 bp followed by E24377A displaying sequences of 2923–2925 bp. One* rrn* of UM 146 and ETECH10407 were of 1762 bp and 1561 bp, respectively (Supplementary Data A.9). Absolutely no link between* rrn* size and pathogenicity could be defined because the variations were random, ATCC8739 being a laboratory culture of fecal origin and UM146 an EIAC strain, E23477A and ETECH10407 of ETEC origin.

Ribosomal genes were found to be distributed across complementary and regular strand with majority of the genomes (46 out of 57) showing segregation in five regular and two complementary strands. All the 23S* rrn* sequences distributed within the regular and complementary strands across respective genomes were individually sorted for MSA to attain an understanding of intragenic variation. Analysis revealed the highest level of mismatches in complementary strands compared with their counterparts (Supplementary Data A.6). The highest level of mismatches mounting to 0.6%, corresponding to 50% of the targeted gene size was perceived in complementary strands of P12b (commensal), E2348/9 (EPEC), and ETEC H10407. The greatest disparity among sequences in regular strand was observed in APEC 01 with 0.03% (88/2903) followed by CFT043 (UPEC) and 042 (EAEC) having 0.016% (~42–51/2903) dissimilarity. 23S ribosomal copies of ED1a (commensal) showed zero percent mismatches in both complementary and regular strands making it an ideal cluster with the highest degree of conservation. Similar values of mismatches were seen in SE11 (Commensal), CB9615 (EPEC), and 0104:H4 2009El-2011 (EAEC). These results indicate that there is no correlation between intragenic diversity of target stretch and pathogenicity of strains. These differences substantiated 23S RNA clusters having microdiverse organization with both conserved and variable stretches within them. Sequevars arising due to this heterogeneity unavoidably lead to biased results, differential amplification, and chimeric molecules. Thus, the method of screening and targeting restriction profile pattern within a region of choice (ribosomal segments) and specific to bacteria of interest demonstrates authentic and simpler practice than routine molecular strategy involving amplification and sequencing.

### 3.2. Barcoding* E. coli* from Its Colony Morphovars: ARDRA of 23S P1_880_ with* Bfa* I

**Figure 2:**
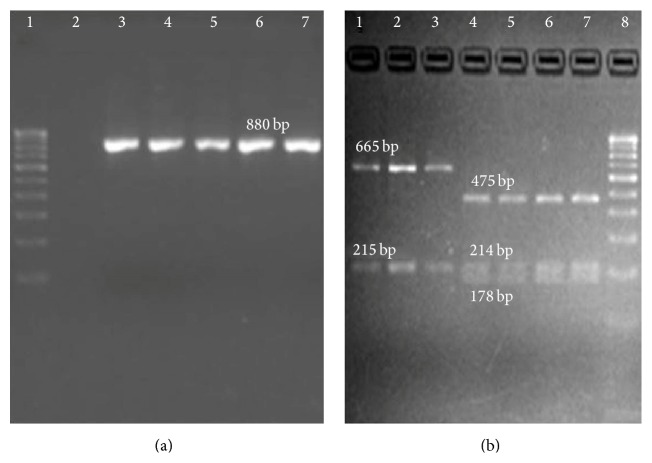
(a) Broad Range PCR of 23S P1_880_ with genomic DNA isolated from standard strains. Lane 1: 100 bp ladder; Lane 2: blank; Lane 3:* E. coli*; Lane 4:* C*.* freundii*; Lane 5:* C. koseri;* Lane 6:* K. pneumonia*; Lane 7:* E. aerogenes*. PCR product of single representative of* E. coli* was loaded. (b) ARDRA of 23S P1_880_ amplicons with* Bfa* I: Lanes 1, 2, and 3:* E. coli* (ATCC15223, ATCC25922, and ATCC11775); Lane 4:* C. freundii* (ATCC8090); Lane 5:* C. koseri* (ATCC27028); Lane 6:* K. pneumonia* (ATCC33495); Lane 7:* E. aerogenes* (ATCC13048); Lane 8: 100 bp ladder.

PCR amplification of the standard strains and isolates of different origin (commensal, urine, and clinical sample) with 23S P1_880_ gave amplicons (880 bp) of high intensity with no primer-dimer formation (Figure 2(a)). Restriction digestion of the amplicons with* Bfa* I gave unique fragments of 665 bp and 215 bp in* E. coli* whereas* C. koseri, E. aerogenes*, and* K. pneumoniae* each gave fragments of 475 bp, 214 bp, and 189 bp, respectively (Figure 2(b)). The genotypic strategy proved superior to biochemical characterization taking into consideration all the possible positive, negative, and false positive and negative results across 25 isolates each from clinical, commensal, and environmental origin (Supplementary Data A.7). 18 (72%) of clinical, 22 (88%) of commensal, and all the 25 (100%) environment isolates showed distinct restriction pattern like that of* E. coli*. BLAST genome search across 931 bacterial families verified the specificity of primers and restriction preference for* Bfa* I. The primers showed exclusive binding only with Enterobacteriaceae family (Supplementary Data A.8). Among the various strains in the family only* Shigella* spp.,* Salmonella enterica* (*Arizona*), and* Proteus mirabilus* exhibited similar restriction preference to that of* E. coli*. In spite of* Shigella* spp. being taxa in disguise of* E. coli* [37] they can be easily screened using selective medium because of no lactose fermentation [38] whereas* Salmonella enterica* and* Proteus mirabilis* showed amplicon heterogeneity of 1073 bp and 880 bp during amplification thus making it easy to avoid erroneous conclusions and misinterpretation.

### 3.3. Categorizing* E. coli* Strains Using ARDRA of 23S P2_682_ with* Hae* III

A detailed analysis of the* Hae* III profile across 399 sequences [Supplementary Data A.9] of ribosomal segments of* E. coli* indicated four possible profiles, types I, II, III, and IV, with varying degree of predominance within these segments. The rest of the candidate strains showed type III patterns but types I and IV were exclusive for* E. coli* strains. Majority of genomes (*n* = 32) as in 15 nonpathogenic and 17 pathogenic strains showed type I profiles (57%) followed by type III (35%) predominating in 15 strains. Six strains showed type IV pattern (10%) of which four were B strains. Type I groups represent* E. coli* strains that lacked recognition elements due to change at 2205th and 2161st base with respect to 23S rDNA or with reference to amplicons at 207th or 249th base positions. Type IV strains are contrary to type I with intact recognition sequence at both of these sites. Strains with type II profiles lacked 2161st (249th) cut site and type III lacked the 2205th (207th) cut positions, respectively. Type II was the rarest with its patterns observed to be ambiguous in certain* rrn* segments.

**Figure 3:**
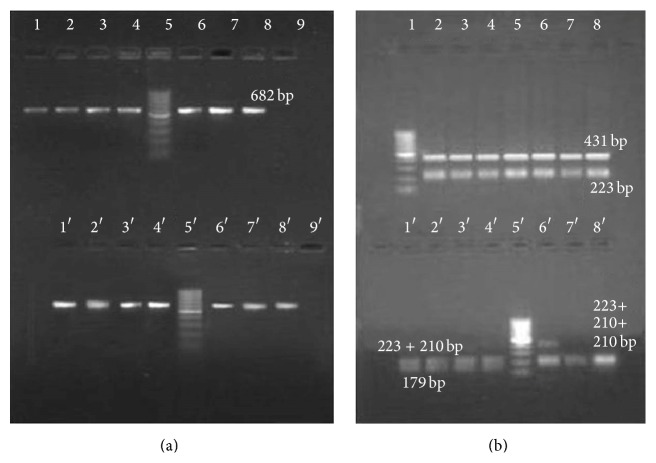
(a) Broad range PCR of 23S P2_682_ with genomic DNA isolated from standard strains of* E. coli*. The upper row comprises Lane 1: K-12 strains; Lane 2: derivatives of K-12 strains; Lane 3: DHI strain; Lane 4: strain C1; Lane 6: Crook's strain; Lane 7: Walker's strain; Lane 8: descendent of W; Lane 9: blank. The lower row comprises Lane 1′: B strain; Lane 2′: BL21 strain; Lane 3′: BL21DE3; Lane 4′: BL21DE3Lys5 (B3); Lane 6′:* E. coli (*ATCC 15223, commensal isolate); Lane 7′:* E. coli* (ATCC 11775; urine sample); Lane 8′:* E. coli* (ATCC 25922, clinical). Lanes 5/5′ and Lanes 9/9′ in both rows are 100 bp ladder and blank, respectively. (b) ARDRA of 23S P2_682_ amplicons with* Hae* III: Lane 1 of both rows comprises 100 bp ladder. The digested products are loaded as per the order given in Figure 2(a).

Heterogenicity in few genomes was noted; two* rrn* among seven repeats of W (NC_017685.1, each in regular and complementary strand) and KO11FL (NC_017660.1, each in regular and complementary strand) and one in KO11FL (NC_016 902.1, regular) showed type II profile in spite of type I being their primary pattern. One among seven of the B strains REL 606 (NC_012967.1, regular), BL21-Gold (NC_012947.1, complementary), BL21DE3 (NC_012971.2, regular), and BL21DE3 (NC_012892.2, regular) showed type I profiles apart from being prevalent with type IV pattern. Similarly, two* rrn* among NRG 873C (NC_017634.1, each in regular and complementary strand) and LF82 (NC_011993.1, each in regular and complementary strand) and one in CFT073 (NC_004431.1, regular) showed type I pattern while exhibiting type III pattern predominance. APEC01 (NC_008563.1) being prime in type III showed one* rrn* segment of type I and type IV (both in regular strand).* E. coli* strain 042 (NC_017626.1) displayed preponderance in type I pattern but three* rrn* (two regular and one complementary) copies exhibited type III pattern. One of the regular* rrn* elements in LF82 showed type IV pattern. The fragmentation pattern obtained by ARDRA of 23S P2_682_ with* Hae* III in the standard samples showed that all K-12, C, W, Crooks, and their respective descendants exhibited type I profiles with type IV profiles being exclusively presented by B strains (Figures 3(a) and 3(b)).

Among the isolates, 21 out of 22 commensal and 16 of 18 clinical samples showed type I pattern; heterogenicity of patterns was not observed. These results reaffirmed predominance (93%) of type I in nature. Multisequence typing has shown commensal strains to be categorized primarily under group A or group B1 whereas groups B2, D, and E comprise generally pathogenic strains; K-12, B, and Crooks strain come under phylogroup A whereas strain W is a B1 phylogroup.

### 3.4. Local Alignment of 23S* rrn Hae* III Profiles versus Global Alignment to Establish Clonality in* E. coli*

**Figure 4:**
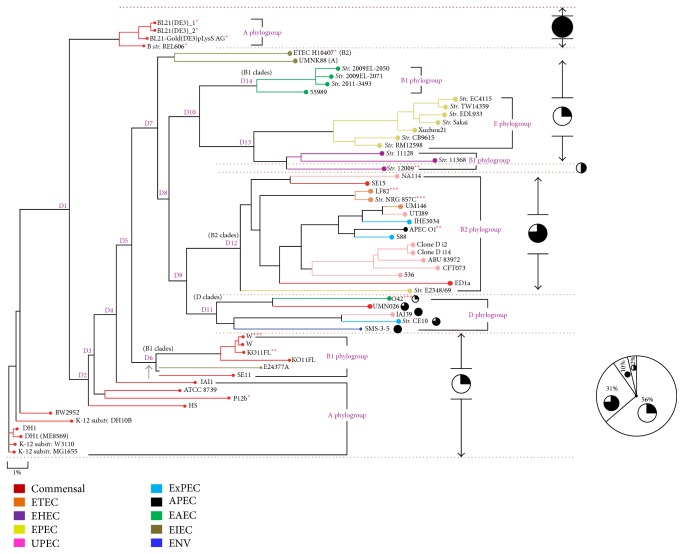
Analysis of distribution of* Hae* III profiles, phylogroups, and pathovars within the global alignment dendogram construct comprising 57* E. coli* genomes. The dendogram of the whole genome sequence of 57 genomes was obtained from the NCBI web site for* E. coli* and was redrawn using Photoshop CS6 for colour coordination for pathogenic and nonpathogenic strains and inputting the clonal groups and phylogroups (http://www.ncbi.nlm.nih.gov/genome/?term=escherichia+coli). Among the 57 genomes, 32 were of type I (56%), 18 were of type III (31%), and 6 and 1 were of type IV (10%) and type II (2%), respectively, showing that type I is predominant and type II the rarest. To correlate between the phylogroups,* Hae* III profiles, and pathovars, the lineages were considered as clades derived from various divergence point (D) during the course of evolution.

A number of genes that are of core, dispensable, and unique in nature make up for pangenome; the latter two are gained due to recombinational or transpositional events. To obtain similar clustering on comparing whole genome would emphasize the significance of these restriction profiles and help in establishing the clonal nature and predominance of a single clone. Hence dendogram constructs with 682 bp amplicon from a representative of each restriction profile will show distinct clustering of these profiles due to the fact that a small subset of segments within the whole genome was considered. The global alignment of whole genome sequences (Figure 4) of the entire 57* E. coli* strains displayed distinct concentrate of type I, III, and IV profiles. An intrinsic analysis of the spot of divergence and respective clade formations showed interesting and riveting findings. The strains at the ancestral root showed type I pattern and the next divergent node point (D1) displayed formation of two distinct clans of type I and type IV profiles segregated at the maximum distance from one another. Numbering of nodes was done by considering the precedence of branch point at every node. A possible scenario of two additional mutational events that are of transitional nature (A to G) at 2205th and 2161st bases has introduced* Hae* III recognition patterns. D6 gave lineages of B1 phylogroups, D9 giving rise to B2 clades (D12) of type I profiles and D clades with varied* Hae* III patterns (D11). D10 gave rise to two nodes (with strains being type I groups), with one showing B1 phylogroups (D13) and the other being predominant in E phylogroups followed by B1. An almost exemplary corelation between the clonal groups and different pathovars was found with this study. Majority of commensal strains and EIEC and all EAEC and EPEC were shown to be having type I profile (Table 4). All UPEC, ExPEC, APEC, EIEC, and single strain of EPEC and two commensal strains showed type III pattern. B form strains along with a single strain of UPEC and environmental isolate exhibited type IV profiles.

**Figure 5:**
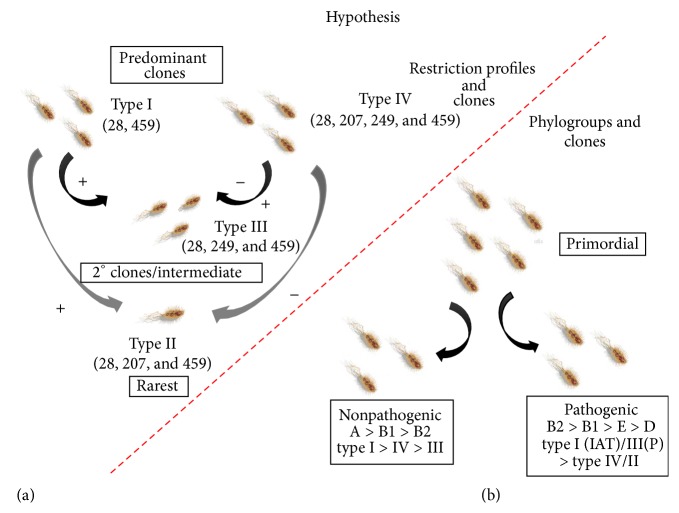
(a) Hypothesis of clonality in* E. coli* strains based on 23S* rrn* restriction profiling. The primordial soup of* E. coli* microbes would have been comprised of two clonal groups, types I and IV coexisting with each other but predominated by type I strains. Over the course of evolution on accumulating mutation as a means for variation, type I or type IV would have given rise to type III by addition (former) or deletion (latter) of recognition sequence. The second scenario could be a rare event because accumulation of two mutations in a conserved region would be difficult. Type II being the rarest would have been evolved in similar basis to type III. (b) Hypothesis of linking phylogroups,* Hae* III profiles, and clonal types in* E. coli*. This diagram illustrates the clonal groups of commensal* E. coli* strains having both A phylogroups (dominant) and B1 phylogroups each being type I profiles. Pathogenic counterparts showed dominancy in type III restriction pattern followed by type I pattern. With respect to phylogroups, pathovars were predominant in B2 and B1 followed by D and E groups.

Similarly, a near-pristine association between phylogroups and* Hae* III profiles was established. Strains coming under phylogroups A were preponderant in type I whereas type IV profiles were exclusively for B forms. Type I profiles were observed in the strains (*n*) in the order of A > B1 > E > D with type III dominant in B2 strains. Phylogroup D showed equal occurrence of type III and type IV fragmentation pattern followed by a single strain with type I profile. These results assisted in enumerating a conjecture which works up a scenario of primordial microbial population where* E. coli* strains had two clonal types (Figure 5(a)) which coexisted together (type I and type IV clones). Of these, type I strains would have been the predominant clone in comparison to type IV based on the dendogram data with maximum gene pool of root strains showing type I. Type III may have been the next predominant clone (an intermediary one) that has evolved either from type I (by addition of a new recognition site) or from type IV (by deletion of one recognition site) strains. In a different scenario maybe all three types coexisted or type III would have given rise to type IV with an additional mutational event. This situation may be hardly convincing as dendogram segregation shows type III strains evolving or present after type IV strains. The clustering also shows that predominance of type III is more than type IV and maybe evolutionary processes/mutation have triggered the increase in type III profiles. On similar line type II clones would have been evolved from type I (addition) or type IV (deletion) but due to the very limited type II restriction profile patterns (rare) as observed from the gene pool data, the chances or approximation of such event is difficult to prove. Based on these results no new insights into phylogroup distribution across candidate strains can be provided; however with respect to the* Hae* III profile an additional angle can be considered. Phylogroups A and B1 are predominant with type I* Hae* III profiles whereas B2 phylogroups exclusively represented type III profile (Figure 5(b)).

An intrinsic metagenomic and pangenomic analysis of most bacterial species exhibited mosaicisms of their inherent blueprint [39]. In such situations, core gene elements shared across the species are limited in comparison to dispensable (flexible) genetic contents that concede uniqueness to a particular strain. These flexible contents help in niche adaptability and give bacteria the ability to infect and invade. A major concern is while diagnosing both true and opportunist pathogenic bacteria, the former causing disease in virtually any susceptible host and the latter invoking diseases in immunocompromised individuals. “Molecular barcoding,” an evolved term which implies the method of allele specific amplification of relatively small DNA sequences or barcodes [40] for spotting a particular species, is a thrust area for microbial diagnostics. A plethora of functional genes ranging from housekeeping to repair are employed for characterizing bacteria of which rRNA is referred to as the gold stretch [41, 42]. Ribosomal segments organized as operon clusters of 16S, 23S, and 5S rDNA and these assemblages are distributed from single to multiple copies across the bacterial genophore [43]. The occurrence of redundancy in maintaining different copies of these genes is not associated with virulence but an in-built ecological strategy that contributes to the particular bacterium with a competitive edge and better chance of survival [44]. A futuristic approach of coupling the technique and results with antibiotic resistance pattern, colony characteristics, and genomic profiles of bacterial strains of both commensal and pathogenic nature can help in monitoring and managing infections in a better manner.

## 4. Conclusion

The causatum of postgenomic era, genomics and proteomics, has enhanced this progress by improvising the techniques used for diagnostics. The strategy of ARDRA utilized in this study was rapid and a cost-effective approach to differentiate* E. coli* from its colony morphovars along with giving a predicament of nature of origin or clonal groups. The mutations reported in this work for detection and establishing clonality, namely, T874A (*Bfa* I), G2161A, and G2205A (*Hae* III), have not been mentioned in any research paper or within the Ribosomal RNA Mutation Database [45] making this finding aboriginal. This basic methodology can be adapted for detection of other bacterial organisms by targeting SNP/s linked to recognition sequences, thereby validating* rrn* sequences as marker for diagnostics. A cumulative approach of detection of unique restriction pattern of a targeted gene and its cross-reference across bacterial families can circumvent changes of false positives.

## Supplementary Material

The description of the supplementary material is with respect to genome name, the serotype, number of plasmids, accession number of genome data, GC content, sequencing center and source of sample for both candidate and non-candidate genomes used for this study has been listed in this spread sheet.

## Figures and Tables

**Table 1:** List of standard strains used in this study.

Strains	MTCC	ATCC	Nature
Barcoding/identification studies			

Noncandidate strains			
*Citrobacter freundii*	1658	8090	Clinical
*Citrobacter koseri*	1657	27028	Blood culture
*Klebsiella pneumonia*	432	33495	Urinary culture
*Enterobacter aerogenes*	111	13048	Sputum

Candidate strains			
	433	15228	Commensal
*Escherichia coli*	4296	19138	Urine
	448	25922	Clinical sample

Diversity/clonality studies			

*Escherichia coli*	1302	—	K12
	2622	10798	Descendent of K12
	1667	—	DH1
	1304	—	C1
	1687	8739	Crooks strain
	448	9637	W
	2692	53846	Descendent of W
	1303	—	B
	1678	—	BL21
	1679	—	BL21DE3
	1680	—	BL21DE3Lys5

*E. coli*, namely, K-12, B, C, Crooks, and W, are commonly used as lab strains. Whole genomic data of these strains except strain C is available (NCBI Genomes).

**Table 2:** Restriction site of *Bfa* I (C/TAG) within 23S *rrlH* of candidate and noncandidates and the restriction profiles with 23S P1_880_.

Restriction profile within *rrl*H					Fragments obtained on ARDRA	
F	E	Ci	K	Ea	Mic.	Fragments (amplicon size)
1.1	**687**	**685**	**685**	**685**	E	665 bp, 215 bp (880 bp)
1.2		**874**	**874**	**874**	C	475 bp, 214 bp, and 189 bp (878 bp)
1.3	1670	1669	1667	1670	K	475 bp, 214 bp, and 189 bp (877 bp)
1.4			2336	2339	Ea	475 bp, 214 bp, and 189 bp (876 bp)
1.5	2652	2653	2650	2654		

Primers were designed against the regions flanking the bold restriction sites.

F: number of fragments; E: *Escherichia coli*; Ci: *Citrobacter koseri*; K: *Klebsiella pneumoniae*; Ea: *Enterobacter aerogenes*; Mic.: microbe.

23S P1_880_-FP(+): 5′-GGCGAAAAGAACCCCGGCGA-3′ (20 nt); 23S P1_880_-RP(−): 5′-AGGGGTCGACTCACCCTGCC-3′ (20 nt).

FP: forward primer; RP: reverse primer. The primer details were obtained from the results of primer blast and restriction profiling pattern was obtained from NEB V2.0. Forward and reverse primers of 23S P1_880_ lie within domains I and III of 23S rRNA secondary structure, respectively, with *Bfa* I sites in domain II.

**Table 3:** Clonal groups based on *Hae* III (GG/CC) profiles within the candidate microbe using 23S P2_682_.

Virtual digest pattern		Types of *Hae* III restriction pattern observed amongst 399 *rrn* sequences of *E. coli *genomes			
F	C	Type I	Type II	Type III	Type IV
1.1	785	Cut site within *rrl*H of candidate *rrn* segments			
1.2	1042	1984	1984	1984	1984
1.3	1116	x	2163	X	2163
1.4	1217	x	X	2205	2205
1.5	1361	2415	2415	2415	2415
1.6	1445	Cut site within the amplicon (682 bp)			
1.7	1907	28	28	28	28
**1.8**	**1984**	x	207	X	207
**1.9**	**2161**	x	X	249	249
1.10	2205	459	459	459	459
1.11	2415	Fragments obtained for each group			
1.12	2839	28 bp, 223 bp, and 431 bp	28 bp, 179 bp, 223 bp, and 252 bp	28 bp, 210 bp, 221 bp, and 223 bp	28 bp, 42 bp, 179 bp, 210 bp, and 223 bp

Primers were designed against the regions flanking the bold restriction sites. F: number of fragments; C: cut sites.

23S P2_682_-FP(+): 5′CCGACCTGCACGAATGGCGT3′ (20 nt).

23S P2_682_-RP(+): 5′CAGTTCTCCAGCGCCCACGG3′ (20 nt).

Forward and reverse primers of 23S P2_682_ lie within domain IV and transition of V-VI of 23S rRNA secondary structure, respectively. First cut sites lie within domain IV whereas the rest lie within domain V.

**Table 4:** Corelation between phylogroups, *Hae* III profiles, and nature of *E. coli *strains (genome data analysis of 57 *E. coli *strains collated from Genbank).

Strain	Type	A	B1	B2	D	E	Total	Out of
Commensal	I	10	5	—	—	—	15	21
Commensal	III	—	—	2	—	—	2	21
Commensal	IV	4	—	—	—	—	4	21
EPEC	I	—	—	—	—	7	7	8
EPEC	III	—	—	1	—	—	1	8
EAEC	I	—	4	—	1	—	5	5
EHEC	I	—	2	—	—	—	2	3
EHEC	II	—	1	—	—	—	1	3
ETEC	I	1	1	1	—	—	3	3
UPEC	III	—	—	7	—	—	7	8
UPEC	IV	—	—	—	1	—	1	8
EIEC	III	—	—	3	—	—	3	3
ExPEC	III	—	—	2	2	—	4	4
APEC	III	—	—	1		—	1	1
ENV	IV	—	—	—	1		1	1

APEC: avian pathogenic; EAEC: enteroaggressive; EHEC: enterohemorrhagic; ETEC: enterotoxigenic; EIEC: enteroinvasive; EPEC: enteropathogenic; ENV: environmental; ExPEC: extraintestinal pathogenic; UPEC: uropathogenic.
